# Cost-effectiveness of antihypertensive deprescribing in primary care: a Markov modelling study using data from the OPTiMISE trial

**DOI:** 10.1161/HYPERTENSIONAHA.121.18726

**Published:** 2022-03-10

**Authors:** Sue Jowett, Shahela Kodabuckus, Gary A Ford, FD Richard Hobbs, Mark Lown, Jonathan Mant, Rupert Payne, Richard J McManus, James P Sheppard, for the OPTiMISE investigators

**Affiliations:** 1 Institute of Applied Health Research, University of Birmingham, Birmingham, UK; 2 Oxford University Hospitals NHS Foundation Trust and University of Oxford, Oxford, UK; 3 Nuffield Department of Primary Care Health Sciences, University of Oxford, Oxford, UK; 4 Primary Care Research Centre, University of Southampton, Southampton, UK; 5 Primary Care Unit, Department of Public Health & Primary Care, University of Cambridge, Cambridge, UK; 6 Population Health Sciences, University of Bristol, Bristol, UK

**Keywords:** blood pressure, medication withdrawal, hypertension, older adults, general practice, cost utility analysis

## Abstract

**Background:**

Deprescribing of antihypertensive medications for older patients with normal blood pressure is recommended by some clinical guidelines, where the potential harms of treatment may outweigh the benefits. This study aimed to assess the cost-effectiveness of this approach.

**Methods:**

A Markov patient-level simulation was undertaken to model the effect of withdrawing one antihypertensive compared to usual care, over a life-time horizon. Model population characteristics were estimated using data from the OPTiMISE antihypertensive deprescribing trial and the effects of blood pressure changes on outcomes were derived from the literature. Health-related quality of life was modelled in Quality-Adjusted Life Years (QALYs) and presented as costs per QALY gained.

**Results:**

In the base-case analysis, medication reduction resulted in lower costs than usual care (mean difference £185), but also lower QALYs (mean difference 0.062) per patient over a life-time horizon. Usual care was cost-effective at £2,975 per QALY gained (more costly, but more effective). Medication reduction resulted more heart failure and stroke/TIA events but fewer adverse events. Medication reduction may be the preferred strategy at a willingness-to-pay of £20,000/QALY, where the baseline absolute risk of serious drug-related adverse events was ≥7.7% a year (compared to 1.7% in the base-case).

**Conclusions:**

Although there was uncertainty around many of the assumptions underpinning this model, these findings suggest that antihypertensive medication reduction should not be attempted in many older patients with controlled systolic blood pressure. For populations at high risk of adverse effects, deprescribing may be beneficial, but a targeted approach would be required in routine practice.

## Introduction

Hypertension is the leading risk factor for cardiovascular disease,^1^ the commonest cause of morbidity and mortality worldwide.^2^ Antihypertensive treatment has been shown to be very effective at preventing cardiovascular disease (CVD) across many different populations, including those with advancing age.^3, 4^ However, most randomised controlled trials focusing on older people^5, 6^ do not include those patients with significant frailty and multi-morbidity who are prescribed many medications to treat their conditions.^7^ As a result, clinical guidelines^8, 9^ recommend caution when prescribing antihypertensive treatment in these older adults, due to a lack of evidence on efficacy and concerns about the potential for drug related harm.^10^

Increasingly, deprescribing of antihypertensive medications is being encouraged in patients with controlled blood pressure,^11, 12^ where the potential harms of treatment^10^ may outweigh the benefits. It is also seen as a mechanism to reduce polypharmacy, since the most common co-morbidity in older people is hypertension^13^ and most patients will need multiple antihypertensive medications to control their blood pressure.^14^ Indeed, it has been suggested that ‘deprescribing’ treatment prescriptions which no longer provide benefit could be cost-saving for healthcare providers.^15^ However, there is very little evidence to support the practice of deprescribing antihypertensives.^16^

The OPtimising Treatment for MIld Systolic hypertension in the Elderly (OPTiMISE) trial sought to address this evidence gap through a randomised, open label, non-inferiority trial of antihypertensive deprescribing (withdrawal of one antihypertensive) versus usual care.^17^ In 569 participants aged 80 years or older, antihypertensive deprescribing was shown to be possible with no difference in the proportion of participants with controlled systolic blood pressure (<150 mmHg) between groups at 12-week follow-up. There were also no differences in serious adverse events or health-related quality of life, although blood pressure did increase modestly (3/2 mmHg) in the deprescribing group.^17^ Whilst this trial suggested that antihypertensive deprescribing was safe in the short-term, the long-term impacts on clinical outcomes remain unknown, as do the cost implications of this strategy if it were to be adopted in routine clinical practice.

The present study aimed to extrapolate results from the OPTiMISE trial to assess the longer-term cost-effectiveness of antihypertensive deprescribing from a National Health Service (NHS)/Personal Social Services (PSS) perspective, using a Markov model with individual patient level simulation.

## Methods

The data that support the findings of this study are available from the corresponding author upon reasonable request.

### Study design

A Markov patient-level simulation was undertaken in TreeAge 2019 (TreeAge Software, Inc., Williamstown, MA, USA) to model the two treatment strategies (usual care and withdrawal of one antihypertensive agent). This type of Markov model tracks the costs and consequences of individual patients passing through the model, with characteristics (taken from OPTiMISE patient-level data)^17^ free to vary between patients. The model was run over a life-time (maximum of 20 years) time horizon to capture all relevant long-term costs and consequences, with a three month time cycle.

### Patient level data collection

Full details of the OPTiMISE trial have been published elsewhere.^17, 18^ Briefly, this was a randomised controlled trial assessing a strategy of antihypertensive medication reduction (withdrawal of one drug) compared with usual care where no medication changes were mandated. Eligible patients were aged ≥80 years with systolic blood pressure <150mmHg and receiving ≥2 antihypertensive medications, whose primary care physician considered them appropriate for medication reduction due to increasing frailty and/or multi-morbidity.

The primary outcome of the trial was to determine whether a reduction in medication could be achieved with a proportion of participants maintaining clinically safe blood pressure levels (defined as a systolic blood pressure <150mmHg) that was non-inferior to that achieved by the usual care group, over 12-weeks follow-up. Data were collected on prescribed antihypertensives, quality of life (EQ-5D-5L), number of cardiovascular comorbidities and all variables required for the calculation of 10-year cardiovascular risk using the QRisk2 algorithm.^19^

### Study population

Patients in the model had characteristics (age, sex, cardiovascular risk) created by randomly sampling the trial patient-level data by means of a uniform distribution. These characteristics affected their probability of subsequent model events. The model was run with a large number of simulated patients (100,000) to account for inter-patient variability and to adequately model a representative clinical population.

### Model comparators and costs

In keeping with the original trial intervention, patients receiving the medication reduction strategy had a 4-week follow-up safety appointment and treatment was reinstated if systolic blood pressure was found to be above 150 mmHg for more than one week, adverse events occurred or signs of accelerated hypertension developed. Both strategies included the cost of ongoing primary care consultations (assumed to be an average of 0.8 per 3 months [included regardless of whether or not they were related to hypertension management)^20^ and antihypertensive prescriptions (Table S1). The medication reduction strategy also included the cost of the 4-week safety appointment, and an additional visit if treatment was reinstated. Costs of modelled clinical events (detailed in the Model Structure) including initial acute care costs and long-term care were obtained from previously published work, expert opinion and standard reference costs (Table S1).^21-31^ Costs are reported in 2017/2018 prices (reflecting the trial timeframe) and inflated where applicable using the New Health Services Index.^32^

### Model Structure and Assumptions

Within each 3-month time cycle, a patient had a risk of suffering a cardiovascular event, an antihypertensive-related serious or minor adverse event, or death (Figure S1). Possible cardiovascular events were coronary heart disease (stable angina, acute coronary syndrome, myocardial infarction), heart failure, stroke and transient ischemic attack (TIA). Antihypertensive-related adverse events were acute kidney injury, hospitalised and non-hospitalised falls, hypotension, syncope, bradycardia and electrolyte imbalance. Ten-year cardiovascular risk was calculated for each individual patient using the QRisk2 algorithm.^19^ In the absence of robust published estimates in this older population, an assumption of greater CVD risk was applied to those with CVD conditions by applying a multiplier of 1.5, based on expert clinical opinion. The distribution of coronary heart disease and stroke/TIA events was dependent on age and gender^22^ and heart failure risk was dependent on age.^33^ The risk of minor and serious adverse events (serious falls, acute kidney injury) from antihypertensive treatment were obtained from SPRINT data in those aged 75 and over (table 1).^34^

Patients who suffered a non-fatal cardiovascular event or serious antihypertensive-related adverse event transitioned to a post-event health state with an adjusted mortality risk. Additional clinical events or medication changes were not modelled.

The impact of changes in blood pressure was taken from a meta-analysis of blood pressure lowering trials, focussing on patients aged over 80 (table 1).^4^ These were applied as a relative risk, taking into account the mean difference in systolic blood pressure observed in the OPTiMISE trial (3.4 mmHg higher in the intervention group),^17^ using log-linear interpolation. In the base-case analysis, it was assumed that the 12 week differences were maintained over the patient life-time. A half-cycle correction was applied to model costs and outcomes. Future costs and outcomes were discounted at an annual rate of 3.5% as recommended by NICE.^35^ All model assumptions are summarised in Table S2.

### Model Outcomes

Health-related quality of life outcomes were modelled in Quality-Adjusted Life Years (QALYs), taking into account quality of life and survival. Utility scores for health states are detailed in table 1. Initial quality of life was estimated as the overall mean EQ-5D-5L index^36^ at baseline taken from the OPTiMISE trial,^17^ calculated using the NICE-recommended crosswalk algorithm.^37^ Utility values for long-term CVD events and serious adverse effects of treatment were applied multiplicatively to baseline utility scores. Disutilities for TIA and minor side effects were assumed to last for one month and were subtracted from utility scores for one time cycle. Utility decrements for acute kidney injury were applied every 3 months for life. Gender-specific life tables were used to determine the probability of death at different ages, with adjustment to avoid double counting of circulatory deaths.^38, 39^

### Analysis

A cost-utility analysis from an National Health Service/Personal Social Services perspective was undertaken to estimate Incremental Cost-Effectiveness Ratios (ICERs). An ICER was calculated as the difference in costs divided by the difference in QALYs of two strategies, with results presented as cost per QALY gained. The cost-effectiveness of an intervention was considered in relation to the lower NICE threshold of £20,000 per QALY.^40^ Probabilistic Sensitivity Analysis (PSA) was undertaken to assess parameter uncertainty.^41^ Beta distributions were attached to probabilities and utilities, and gamma distributions were attached to costs. Log normal distributions were used for the relative risks associated with the change in systolic blood pressure from the intervention and mortality. The model was run for 1,000 iterations across 100,000 patients and the results are expressed as a Cost-Effectiveness Acceptability Curve (CEAC).^42^ Additional analysis was undertaken to estimate the number of disease events in each category per 100,000 patients.

### Deterministic Sensitivity Analyses

Analyses to evaluate the impact of changing model assumptions and values were undertaken to assess model robustness.^41^ Whilst all parameter values were tested, focus was placed on areas of greatest uncertainty (in the underlying data), which could have the largest impact on the study results. The following scenarios were explored:

1. Threshold analyses examining:
  - the minimum baseline risk of serious adverse events required for usual care to exceed the £20,000/QALY threshold for cost-effectiveness.
  - the minimum additional ‘utility’ required to result in quality of life improvements in those patients reducing medications.
2. Sensitivity analyses examining:
  - alternative values for the relative risk of cardiovascular and medication-related adverse events (using the upper and lower 95% confidence intervals [table 1] or a relative risk of 1).
  - the effect of halving the risk of all cardiovascular events.
  - using the lower 95% confidence interval of the increase in systolic blood pressure with the intervention (1 mm Hg).
  - the effect of reducing the length of time the difference in blood pressure is sustained (ranging from 1 year to 10 years).
  - the effect of reducing the time horizon to 5 years.
3. Sub-group analyses examining the results by level of frailty^43^ (fit or frail) and number of cardiovascular disease co-morbidities at baseline (none, 1, 2+).

## Results

### Cost-effectiveness of medication reduction

**Figure 1:**
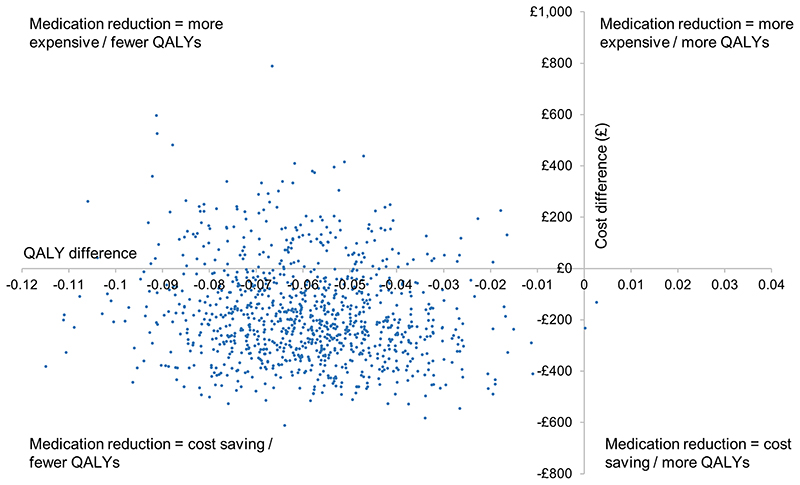
Cost-effectiveness plane for medication reduction versus usual care QALY=quality adjusted life years

**Figure 2:**
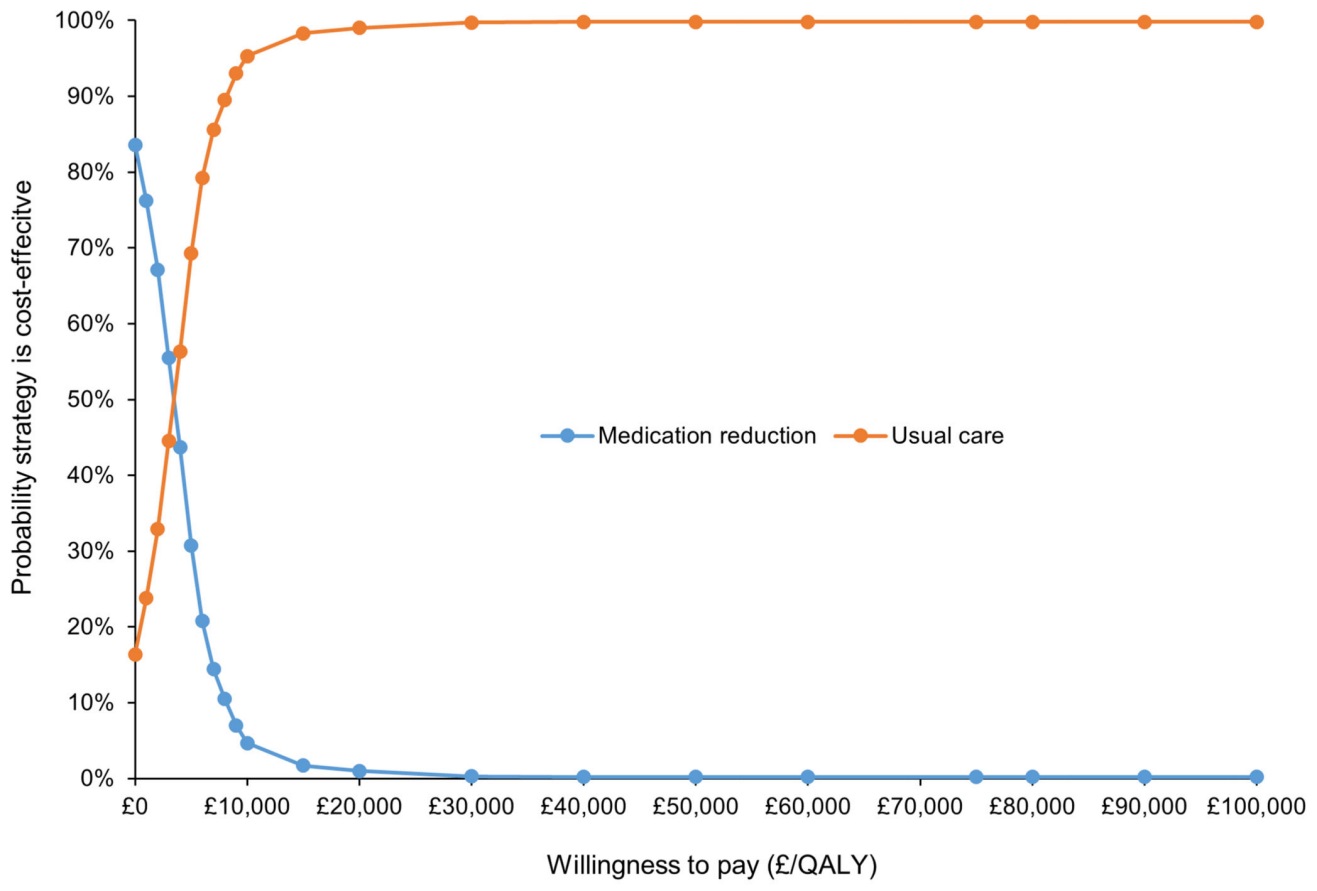
Cost-effectiveness acceptability curve for medication reduction versus usual care Probability that usual care is cost effective at £20,000/QALY=99.0% QALY=quality adjusted life year

In the base-case analysis, medication reduction resulted in lower costs than usual care (mean difference £185), but also lower QALYs (mean difference 0.062) per patient over a life-time time horizon (table 2). The Incremental Cost-Effectiveness Ratio (ICER) for usual care was £2,975 per QALY gained (more costly, but more effective), meaning that usual care was highly cost-effective at the £20,000/QALY threshold. The probabilistic sensitivity analyses showed that usual care was the most cost-effective option in 99.0% of iterations at the £20,000/QALY threshold, and 99.7% at £30,000/QALY, with almost all replications of the model in the western half of the plane (fewer QALYs for medication reduction; figures 1 and 2).

Medication reduction was estimated to result in an increase in the number of heart failure, stroke and TIA events, with between 684-2,739 events occurring per 100,000 population over the life-time (20 year) time horizon (table 3). However, medication reduction was associated with fewer adverse events and coronary heart disease events (due to competing risks where patients were more likely to die before experiencing a CHD event) (table 3).

### Sensitivity analyses

Using a willingness-to-pay of £20,000/QALY in the threshold analyses, medication reduction may be the preferred strategy (as the ICER for usual care exceeds £20,000/QALY), where the baseline absolute risk of serious drug-related adverse events was greater than 7.7% a year for each individual in the model (compared with the base-case value of 1.7%; table 2).

Additional threshold analyses demonstrated that patients had to gain more than 0.017 of utility per year from having their medication reduced (compared with the base-case value of 0) for this intervention to become the preferred strategy (table 2). Both analyses assume that decision makers are willing to forgo small QALY gains in order to reduce costs.

Assuming medication reduction conferred no additional risk (RR=1) for cardiovascular disease simultaneously resulted in usual care no longer being cost-effective, with an ICER of £178,631 per QALY (Table S3). Usual care was still cost-effective when applying the upper and lower 95% confidence intervals of the relative risks of cardiovascular events. Applying the same approach for the adverse events did not change the findings of the primary analysis and in some cases usual care became dominant (Table S3).

When the model time horizon was reduced to 5 years, maintaining antihypertensive prescription (usual care) remained cost-effective. The results were also robust when reducing the timeframe of the effect of the intervention (in terms of increased blood pressure) from life-time to 1 year through to 10 years, halving absolute cardiovascular risk, and when using the lower 95% confidence interval of the observed systolic blood pressure change (Table S4). Usual care was also estimated to be cost-effective in subgroup analyses by frailty and number of cardiovascular conditions present at baseline (Table S5). Sensitivity analysis examining the remaining parameter values had no effect on the model findings.

## Discussion

### Main findings

The primary finding of this study was that usual care, compared with antihypertensive deprescribing, was more expensive (due to higher medication costs) but results in more QALYs, and has an ICER of £2,975 per QALY. This indicates that usual care of continuation of antihypertensive drugs is highly cost-effective compared to deprescribing. The lower QALYs associated with the antihypertensive deprescribing strategy occurred due to a projected increase in cardiovascular events (particularly heart failure) caused by a modest sustained increase in systolic blood pressure. Antihypertensive deprescribing was only the preferred strategy when patients were assumed to have a high baseline risk of serious adverse events (e.g. were at high risk of falling or experiencing acute kidney injury in the next year).

Many of the model inputs had considerable uncertainty or required assumptions to be made, due to a lack of evidence in this older population. Based on currently available data, these findings suggest that antihypertensive medication reduction should not be attempted in most older patients with controlled systolic blood pressure. In some specific populations at particularly high risk of adverse drug events, antihypertensive deprescribing may carry some benefits so a targeted approach may be needed if deprescribing is to be adopted in routine clinical practice.

### Strengths and weaknesses

The present analyses were informed by robust data from a pragmatic randomised controlled trial comparing antihypertensive deprescribing with usual care in a primary care setting. Participants recruited to this trial were representative of the general population aged 80 years and older registered at practices in primary care.^17^ This trial was limited to just 12 weeks of follow-up, meaning that the long-term effects of antihypertensive deprescribing had to be modelled on the basis of observed differences in blood pressure. For the base case analysis, such differences were assumed to be sustained over a lifetime which may not reflect experience in routine practice, although sensitivity analyses shortening the period in which a blood pressure difference existed from 1-10 years did not affect the primary findings of the analysis. This short period of follow-up in the trial meant that estimates of treatment safety and efficacy had to be taken from previous treatment *intensification* trials which are likely (and by design of OPTiMISE) to have recruited a different population to that considered for *deprescribing*.^7, 10^ Estimates of cardiovascular disease risk (which drove the observed differences in QALYs) were based on the best available cardiovascular risk score (QRISK2), which was not developed or validated for individuals aged 85 years or older.^19^ Also, whilst the OPTiMISE trial recruited a population of patients similar to the general older population in primary care,^7^ based on the sample size of the trial there may be some uncertainty around some of the parameters included in the model such as age and baseline cardiovascular risk. Changing these values in a sensitivity analysis did not alter the primary findings.

Ninety-eight percent of OPTiMISE trial participants were living with multiple long-term conditions which could carry competing risks eclipsing future cardiovascular disease events. These could not be taken into account in the present analysis due to a lack of evidence. The present model was complex, requiring a number of assumptions related to the risk of CVD and adverse events for which there is little evidence in this population. This meant it was not possible to add further complexity relating to treatment changes following cardiovascular events, terminal care costs or the impact of recurring events which often occur in real world practice. Such uncertainty, and reliance on data from antihypertensive *intensification* trials may have favoured cost-effectiveness of the usual care strategy.

### Findings in the context of existing literature

To our knowledge, this is the first study to examine the cost-effectiveness of antihypertensive deprescribing in older adults aged 80 years and above. Indeed, few studies have examined the cost-effectiveness of deprescribing of any medication classes in routine clinical practice.^44, 45^ Two analyses based on data from the Developing Pharmacist-Led Research to Educate and Sensitize Community Residents to the Inappropriate Prescriptions Burden in the Elderly (D-PRESCRIBE) trial^46^ examined the cost-effectiveness of nonsteroidal anti-inflammatory drugs (NSAID)^44^ and sedatives.^45^ In contrast to the present analyses, these studies found deprescribing of these medications to be a cost-effective intervention, both in terms of saving money and increasing health related quality of life. Although our analysis found antihypertensive deprescribing to be cost saving too, it is possible that the disutility from adverse events related to NSAID and sedative prescribing is higher than that from antihypertensives, resulting in fewer QALYs gained from stopping antihypertensive treatment. This was supported by sensitivity analyses which suggested that an increasing disutility associated with antihypertensive treatment prescription would have resulted in deprescribing becoming preferred strategy. However, such a gain was not observed in the original trial over 3 months of follow-up.^17^ Indeed, there was no significant difference in EQ-5D-5L index between the two trial arms and a change of the magnitude modelled in this sensitivity analysis was outside the 95% confidence interval for the observed difference.

### Implications for clinical practice

Although based on data with some uncertainty, this study suggests that antihypertensive deprescribing may not be cost-effective in older patients aged 80 years and older, and therefore should not be attempted in patients with controlled systolic blood pressure as a routine policy. This is important for guideline and policy makers, who are increasingly encouraging physicians to think about deprescribing chronic medications where the benefits of treatment no longer outweigh the harms.^11, 47, 48^ Sensitivity analyses conducted here were able to identify scenarios where this might occur, notably, in those with a high risk of medication related adverse events. However, it is currently difficult to determine who these patients might be in routine practice. For other treatments, such as anticoagulants, tools exist which can help physicians quantify an individual’s risk of bleeding which may be increased by treatment.^49^ Similar tools predicting adverse events related to antihypertensive treatment would help target deprescribing at those most likely to benefit, although this requires further research. In the interim, for physicians wishing to reduce antihypertensives prescriptions in older patients under their care, tools such as the electronic frailty index^43^ or QAdmissions score^50^ may be considered as a proxy to determine higher risk patients.

## Perspectives

The present analysis found that deprescribing of antihypertensive medication in older adults was cost saving, but resulted in fewer quality adjusted life years gained when compared to usual care. Although sensitivity analyses suggested that such a strategy may be preferred when targeted at individuals at high risk of adverse events, the lack of robust data regarding the underlying risk in this population, and the long-term effects of deprescribing preclude firm recommendations being drawn. Whilst reducing polypharmacy in the elderly may still be a desirable policy, these data suggest that it may be better to attempt withdrawal of medications that don’t reduce major clinical events.

## Supplementary Material

Graphical Abstract

Supplemental Material

## Figures and Tables

**Table 1:** Model Parameters

Parameter	Model estimate	Source
**Patient characteristics**		
Mean age in years	84.8	Sheppard *et al.,* 2020^17^
Sex (% male)	51.5%	as above
No previous CVD	42.9%	as above
1 previous CVD event	29.5%	
2+ previous CVD events	27.6%	
Systolic BP increase (mm Hg) at 12 weeks compared with Usual Care	3.4 (95% CI 1.0 to 5.8)	as above
Proportion maintaining reduced treatment reduction at 12 weeks	66.3%	as above
**Mortality and risk of cardiovascular disease**		
Probability of non-cardiovascular death	Age and sex dependent	England and Wales 2016-2018 lifetables without CVD death^38, 39^
10 year CVD risk (QRISK2): Range	Patient specific	Sheppard *et al.,* 2020;^17^ QRisk2^19^
Ratio of 10 year CVD risk CHD:Cerebrovascular	50:50	Assumption
Proportion of cerebrovascular events (stroke, TIA)	M, 75-84: 81.1%, 18.9% M, 85+: 95.6%, 4.4% F, 75-84: 82.6%, 17.4% F, 85+: 85.2%, 14.8%	Ward *et al.,* 2007^22^
Proportion of CHD events (Myocardial Infarction, Acute Coronary Syndrome, Stable Angina)	M, 75-84: 37.2%, 18.7%, 44.1% M, 85+: 37.5%, 19.4%, 43.1% F, 75-84: 35.8%, 11.9%, 52.3% F, 85+: 37.7%, 10.9%, 51.3%	as above
1-year risk of Heart failure event	80-84: 2.23% 85-89: 3.58% 90+: 5.36%	Conrad *et al.,* 2018^33^
1-year risk of SAEs related to antihypertensives	1.74%	Williamson *et al.,* 2016^34^
Ratio of serious fall:AKI	0.52:0.48	as above
1-year risk of non-serious adverse event	13.7%	as above
**Relative risks with a reduction in medication**		
Coronary heart disease	1.009 (95% CI 0.896-1.135)	Thomopoulos *et al.,* 2018^4^
Stroke/TIA	1.108 (95% CI 1.047-1.177)	as above
Heart failure	1.290 (95% CI 1.134-1.472)	as above
Serious fall/AKI	0.685 (95% CI 0.343-1.366)	as above
Minor adverse events	0.685 (95% CI 0.343-1.366)	as above
**Standardized Mortality Rate (SMR)**		
Myocardial infarction	2.68	Brønnum-Hansen *et al.*, 2001^51^
Acute coronary syndrome	2.19	NICE guidelines, 2010^52^
Stable angina	1.95	Rosengren *et al.,* 1998^53^
Stroke	2.72	Brønnum-Hansen *et al.*, 2001^51^
Transient ischemic attack	1.40	Dennis *et al.,* 1990^54^
Heart failure	2.17	de Guili *et al.,* 2005^55^
Serious fall (hip fracture)	1.49	Finnes *et al.,* 2013^56^
Acute kidney injury	1.18	Bihorac *et al.,* 2009^57^
**Quality of life multipliers**		
Utility for initial health state (no events)	0.769	Sheppard *et al.,* 2020^17^
Stroke	0.629	Ward *et al.,* 2007^22^
Myocardial infarction	0.778	Jiang and You, 2017^58^
Acute coronary syndrome	0.77	Ward *et al.,* 2007^22^
Stable angina	0.88	as above
Heart failure	0.68	Cooper *et al.,* 2008^59^
Serious fall	0.797	Hiligsmann *et al.,* 2008^60^
**Quality of life decrements**	**Annual decrement**	
Transient ischemic attack	0.103	Meckley *et al.,* 2010^61^
Acute kidney injury	0.15	Nisula *et al.,* 2013^62^
Hypotension	0.0290	Ademi *et al.,* 2017^63^
Syncope	0.1	Bress *et al.,* 2017^64^
Bradycardia	0.1	as above
Electrolyte abnormalities	0.1	as above
Non-serious fall	0.1	as above

BP=blood pressure; CVD=cardiovascular; CHD=coronary heart disease; SAE=serious adverse event; TIA=Transient Ischaemic Attack; AKI=Acute kidney injury; NICE= National Institute for Health and Care Excellence;

**Table 2:** Results of base-case and threshold cost-effectiveness analyses

Analysis	Strategy	Costs per patient	Incremental cost	QALYs gained	Incremental QALYs	ICER (£/QALY)	Interpretation
**Base-case analysis**	Reduced medication	£4,560		3.343			Usual care is cost-effective. The reduced medication strategy is not cost-effective (cost savings not worth loss of QALYs)
	Usual Care	£4,745	£185	3.405	0.062	2,975	
**Threshold analysis:** Absolute risk of SAEs = 7.7% per year* Willingness to pay = £20,000/QALY	Reduced medication	£7,275		3.301			Usual care no longer the preferred strategy if risk >7.7% per year. Cost savings worth the loss of QALYs with reduced medication.
	Usual Care	£8,069	£794	3.340	0.039	20,613	
**Threshold analysis:** Additional utility given to patients reducing medication = 0.017 per year. Willingness to pay = £20,000/QALY	Reduced medication	£4,560		3.396			Usual care no longer the preferred strategy if additional utility >0.017 per year Cost savings worth the loss of QALYs with reduced medication.
	Usual Care	£4,745	£185	3.405	0.009	21,302	

QALYs: Quality Adjusted Life Years; ICER: Incremental Cost-Effectiveness Ratio

* Absolute risk of Serious Adverse Events (SAEs) in the base-case was 1.74% per year

**Table 3:** Estimated incidence of outcome events in the base-case analysis over the life-time time horizon

Outcome event type	Outcome events per 100,000 patients		
	Medication reduction	Usual care	Difference between groups*
Heart failure	22,160	19,421	2,739
Coronary heart disease	18,177	18,606	-429
Stroke/Transient ischemic attack	19,376	18,692	684
Serious drug-related adverse event	4,938	6,376	-1,438
Minor drug-related adverse event	39,859	51,568	-11,709

* Positive integer indicates more events in the medication reduction group

## Novelty and Relevance

### What Is New?

- This is the first study to examine the cost-effectiveness of antihypertensive medication reduction in older adults.
- This analysis found that reducing antihypertensive medication in older adults was cost saving, but resulted in fewer quality adjusted life years gained when compared to usual care.
- Medication reduction was found to be the preferred strategy at a willingness-to-pay of £20,000/QALY only where the baseline absolute risk of serious drug-related adverse events was high (7.7% a year or greater).

### What Is Relevant?

- For most older patients with controlled systolic blood pressure, antihypertensive medication reduction was not a cost-effective treatment strategy.
- In some specific populations at high risk of adverse events, antihypertensive medication reduction may carry potential benefits, so a targeted approach may be needed if this strategy is to be adopted in routine clinical practice.

### Clinical/Pathophysiological Implications?

Despite some uncertainty regarding model inputs, due to a lack of evidence in this older population, these findings suggest that antihypertensive medication reduction should not be attempted in most older patients with controlled systolic blood pressure. Further research is required to understand the risks and benefit of antihypertensive medication reduction in older people at high risk of adverse effects from blood pressure lowering.

## Non-standard abbreviations/acronyms

CVD: cardiovascular disease
CHD: coronary heart disease
TIA: transient ischemic attack
BP: blood pressure
SAE: serious adverse event
AKI: Acute kidney injury
NHS: National Health Service
PSS: Personal Social Services
QALYs: Quality-Adjusted Life Years
ICERs: Incremental Cost-Effectiveness Ratios
CEAC: Cost-Effectiveness Acceptability Curve
NICE: National Institute for Health and Care Excellence;
OPTiMISE: OPtimising Treatment for MIld Systolic hypertension in the Elderly trial
SPRINT: Systolic blood Pressure Intervention Trial
D-PRESCRIBE: Developing Pharmacist-led Research to Educate and Sensitize Community Residents to the Inappropriate Prescriptions Burden in the Elderly trial
NSAID: nonsteroidal anti-inflammatory drugs
