# Supplementary figures and images for: Cost-effectiveness of antihypertensive deprescribing in primary care: a Markov modelling study using data from the OPTiMISE trial
Source: Hypertension. Author manuscript; Available in PMC 2022 May 1. (PMC8997697; doi:10.1161/HYPERTENSIONAHA.121.18726)

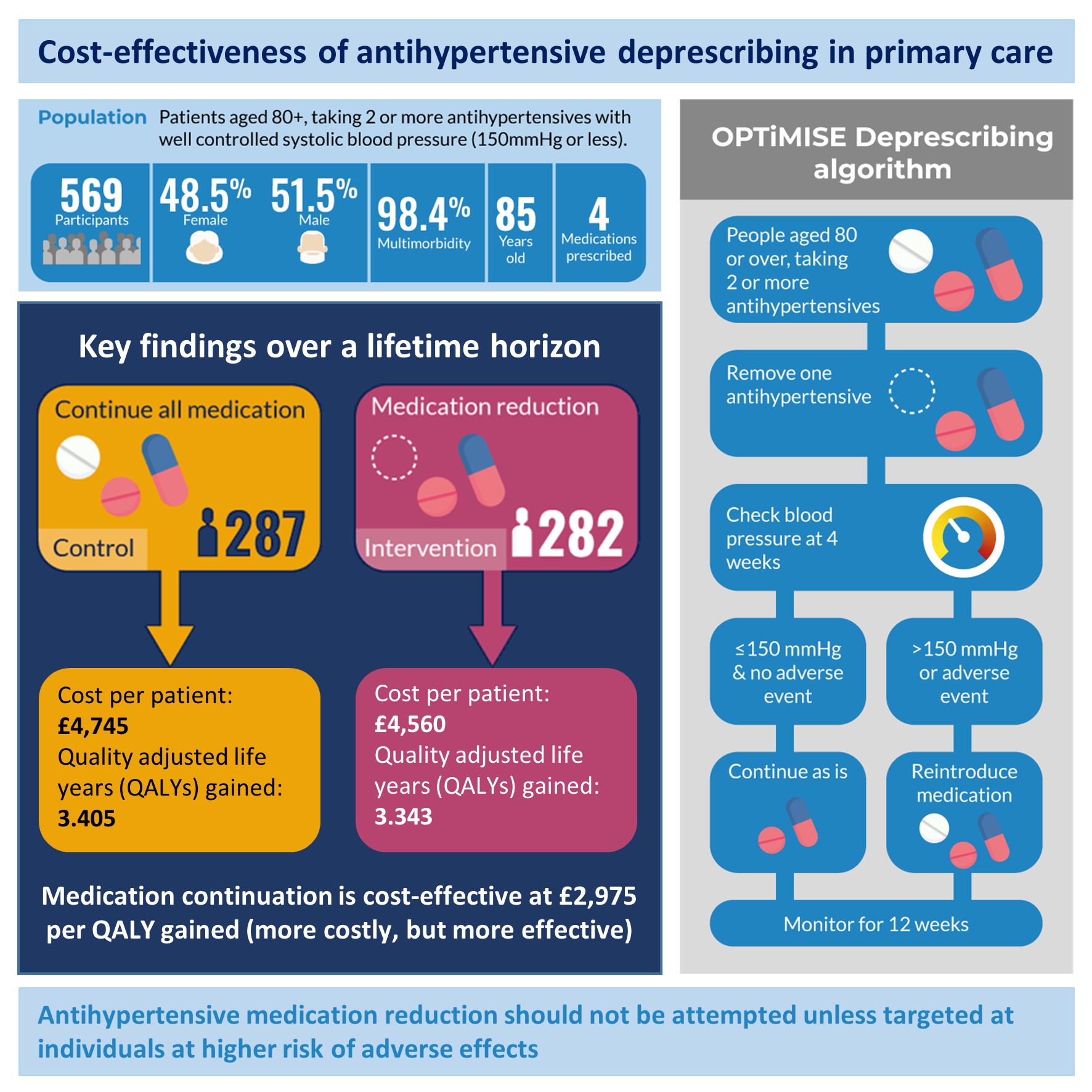

Supplement: Graphical Abstract [file EMS143919-supplement-Graphical_Abstract.jpg]
