# Supplementary material for: Cost-effectiveness of antihypertensive deprescribing in primary care: a Markov modelling study using data from the OPTiMISE trial
Source: Hypertension. Author manuscript; Available in PMC 2022 May 1. (PMC8997697; doi:10.1161/HYPERTENSIONAHA.121.18726)
Supplement: Supplemental Material [file EMS143919-supplement-Supplemental_Material.docx]

# Cost-effectiveness of antihypertensive deprescribing in primary care: a Markov modelling study using data from the OPTiMISE trial

**Supplementary appendix**

Sue Jowett, *PhD,*^1^ Shahela Kodabuckus, *MSc,*^1^ Gary A Ford, *FMedSci,*^2^ FD Richard Hobbs, *FMedSci,*^3^ Mark Lown, *PhD,*^4^ Jonathan Mant, *MD,*^5^ Rupert Payne, *PhD,*^6^ Richard J McManus, *PhD,*^3^ and James P Sheppard, *PhD,*^3^

^1^Institute of Applied Health Research, University of Birmingham, Birmingham, UK

^2^Oxford University Hospitals NHS Foundation Trust and University of Oxford, Oxford, UK

^3^Nuffield Department of Primary Care Health Sciences, University of Oxford, Oxford, UK

^4^Primary Care Research Centre, University of Southampton, Southampton, UK

^5^Primary Care Unit, Department of Public Health & Primary Care, University of Cambridge, Cambridge, UK

^6^Population Health Sciences, University of Bristol, Bristol, UK

**Table of contents**

1. **OPTiMISE investigators**
2. **References**
3. **Table S1.** Model costs
4. **Table S2.** Model assumptions
5. **Table S3.** Sensitivity analysis showing effect varying the relative risk associated with outcome events on the estimated incremental cost effectiveness ratio (ICER)
6. **Table S4.** Sensitivity analyses showing the effect of assuming different effects of blood pressure and absolute cardiovascular risk on the estimated incremental cost effectiveness ratio (ICER)
7. **Table S5.** Sub-group analyses showing the incremental cost effectiveness ratio (ICER) by baseline frailty and cardiovascular co-morbidities.
8. **Figure S1.** Markov model structure

**OPTiMISE investigators**

**Core trial team**

James P. Sheppard, Jenni Burt, Mark Lown, Eleanor Temple, Rebecca Lowe, Rosalyn Fraser, Julie Allen, Gary A Ford, Carl Heneghan, F. D. Richard Hobbs, Sue Jowett, Shahela Kodabuckus, Paul Little, Jonathan Mant, Jill Mollison, Rupert A. Payne, Marney Williams, Ly-Mee Yu, Richard J. McManus

**Coordinating centre (Oxford Primary Care Clinical Trials Unit)**

Alecia Nickless, Christopher Lovekin, David Judge, David Watt, Hannah Ashby, Hannah Swayze, Lazarina Engonidou, Sadie Kelly, Bethany Dimmet (Cambridge), Sarah Oliver (Southampton).

**Participating National Institute for Health Research Clinical Research Networks and staff**

*Thames Valley and South Midlands*

Debbie Kelly, Lydia Owen, Diane Lonsdale, Claire Winch, Sarah Wytrykowski, Katherine Priddis

*Wessex*

Kerry Gunner, Clare Grocutt, Julie Kennedy, Vivian Sparshott

*Eastern*

Clare Fletcher, Jenny Johnson, Kirsti Withington, Marie Corcoran

*West Midlands*

Claire Brown, Elaine Butcher, Eleanor Hoverd, Pauline Darbyshire, Sarah Joshi, Susan Zhao, Tracey Davenport, Andrea Isaew, Julie Timmins, Azaria Ballintine, Somi Spannuth

*West* *of* *England*

Amy Herbert, Bethan Rees, Rwth Leach, Rose Hawkins, Emma Jennings, Lara Peniket, Hanorah Saliba

**Participating practices and general practitioners**

Ascot Medical Centre (Dr Edward Williams), Berinsfield Health Centre (Dr Jonathan Crawshaw), Bicester Health Centre (Dr Robin Fox), The Boathouse Surgery (Dr Thomas Morgan), Bampton Medical Practice (Dr Peter Grimwade), 27 Beaumont Street Medical Practice (Prof Richard McManus), Botley Medical Centre (Dr Mary Akinola), Broadshires Health Centre (Dr Christine A'Court), The Cedars Surgery (Dr Clare Nieland), Cookham Medical Centre (Dr Kenney Tsoi), Church Street Practice (Dr Matthew Gaw), Eynsham Medical Group (Dr Ian Binnian), Hughenden Valley Surgery (Dr Lynette Hykin), Hollow Way Medical Practice (Dr Debbie Goldman), Jericho Health Centre (Dr Mark O'Shea), King Edward Street Medical Practice (Dr Brian Nicholson), Magnolia House Surgery (Dr Kavil Patel), Medicas Health Limited (Dr Ruth Mason), Milman Road Surgery (Dr Aneeka Bajwa), St Clements Surgery (Dr Hernandez), South Oxford Health Centre (Dr Nick Wooding), Summertown Health Centre (Dr Kyle Knox), Temple Cowley Medical Group (Dr Elisabetta Angeleri-Rand), Chalgrove and Watlington Surgeries (Dr Grace Ding), The Downland Practice (Dr Chloe Evans), The Ivers Practice (Dr Neetul Shah), Thatcham Medical Practice (Dr Sarah Wadsworth), Woodlands Medical Centre (Dr Adam Jones), Wokingham Medical Centre (Dr Zishan Ali), Whaddon Medical Centre (Dr Asim Malik), White Horse Medical Practice (Dr Simon Cartwright), Homewell and Curlew Practice (Dr Helen Whiting), Liphook and Liss Surgery (Dr Anna Lalonde), Vine Medical Group (Dr Olivia Boocock), Wareham Surgery (Dr James Bennett), Badgerswood and Forest Surgery (Dr Helen Sherrell), Lordshill Health Centre (Dr Faycal Elhani), Highcliffe Medical Centre (Dr Zelda Cheng), Cowplain Family Practice (Dr Nicola Millen), Blackthorn Health Centre (Dr Ali Shahsavanpour), Feltwell Surgery (Dr Michael Pullen), Bridge Street Surgery (Dr Clare Hambling), Little St John Street (Dr Gary Taylor), Hoveton and Wroxham Medical Practice (Dr Carsten Dernedde), Wansford and Kings Cliffe Practice (Dr Amrit Takhar), Woolpit Health Centre (Dr Richard West), Shelford Medical Practice (Dr Chris Schramm), St Mary's Surgery (Dr Katrina Young), Bennfield Surgery (Dr Nick Doherty and Dr Suparna Behura), West Heath Primary Care Centre (Dr Gulshan Arora), Westside Medical Centre (Dr Mark Lindsey), Eve Hill Medical Practice (Dr David Shukla), River Brook Medical Centre, (Dr Naresh Chauhan), Park Leys Medical Practice (Dr Rachel Spencer), Manor Court Surgery (Dr Farhana Lockhat), The Marches Surgery (Dr Crispin Fisher), Winyates Health Centre (Dr Vattakkatt Premchand), Avonside Health Centre (Dr Sarah Colliver), Hastings House Surgery (Dr Paul de Cates), Maypole Health Centre (Dr Soong Loy Yap), Eden Court Medical Practice (Dr Naresh Aggarwal), Old Priory Surgery (Dr Mark Grocutt), Courtside Surgery (Dr Vicky Smith), Trowbridge Health Centre (Dr Toby Cookson), Phoenix Surgery (Dr Naomi Vernon), West Walk Surgery (Dr Sam Davies), Winchcombe Medical Centre (Dr Richard Tribley), Mann Cottage Surgery (Dr Cathy Bobrow), Chipping Campden Surgery (Dr Rebecca Zamir)

**Trial Steering Committee**

Prof Tom Robinson (Chair, University of Leicester), Prof Rod Taylor (University of Exeter), Prof Richard Lindley (University of Sydney), Prof Peter Bower (University of Manchester), Ms Margaret Ogden (Patient and public involvement representative), Ms Anita Higham (Patient and public involvement representative)

**Data Monitoring Committee**

Prof John Gladman (Chair, University of Nottingham), Prof Una Martin (University of Birmingham), Dr Martyn Lewis (Keele University)

**References**

1. Curtis L, Burns A. Unit costs of health and social care. 2018

2. Luengo-Fernandez R, Gray AM, Rothwell PM. A population-based study of hospital care costs during 5 years after transient ischemic attack and stroke. *Stroke; a journal of cerebral circulation*. 2012;43:3343-3351

3. Ward S, Lloyd Jones M, Pandor A, Holmes M, Ara R, Ryan A, et al. A systematic review and economic evaluation of statins for the prevention of coronary events. *Health technology assessment (Winchester, England)*. 2007;11:1-160, iii-iv

4. Joint Formulary Committee. *British national formulary*. London: BMJ Group and Pharmaceutical Press; 2018.

5. Palmer S, Sculpher M, Philips Z. A cost-effectiveness model comparing alternative management strategies for the use of glycoprotein iib/iiia antagonists in non-st-elevation acute coronary syndrome. *Technology Assessment Report*. 2002

6. Taylor M, Scuffham PA, Chaplin S, Papo NL. An economic evaluation of valsartan for post-mi patients in the uk who are not suitable for treatment with ace inhibitors. *Value in health : the journal of the International Society for Pharmacoeconomics and Outcomes Research*. 2009;12:459-465

7. Department of Health. National schedule of reference costs - year 2017-2018. 2018

8. National Institute for Health and Care Excellence. Management of stable angina. 2011

9. Griffiths A, Paracha N, Davies A, Branscombe N, Cowie MR, Sculpher M. The cost effectiveness of ivabradine in the treatment of chronic heart failure from the u.K. National health service perspective. *Heart (British Cardiac Society)*. 2014;100:1031-1036

10. National Institute for Health and Care Excellence. Hip fracture: Management. Clinical guideline cg124. . 2011

11. Polinder S, Boyé ND, Mattace-Raso FU, Van der Velde N, Hartholt KA, De Vries OJ, et al. Cost-utility of medication withdrawal in older fallers: Results from the improving medication prescribing to reduce risk of falls (improvefall) trial. *BMC geriatrics*. 2016;16:179

12. Hall PS, Mitchell ED, Smith AF, Cairns DA, Messenger M, Hutchinson M, et al. The future for diagnostic tests of acute kidney injury in critical care: Evidence synthesis, care pathway analysis and research prioritisation. *Health technology assessment (Winchester, England)*. 2018;22:1-274

**Table S1.** Model costs

| Costs |  |  |
| --- | --- | --- |
| Care/clinical event | **Cost per patient** |  |
| Usual Care (3 months) | £50.43 | 0.8 GP consultations and average medication from OPTiMISE (£18.03) |
| Medication reduction  (first 3 months) | £54.97 | 1 GP consultation and average medication from OPTiMISE (£14.47) |
| Medication reduction (subsequent 3 months) | £46.87 | 0.8 GP consultations and average medication from OPTiMISE (£14.47) |
| GP consultation (10 minutes) | £40.50 | Curtis *et al.,* 2018^1^ |
| Nurse consultation  (10 minutes) | £7.00 | as above |
| Acute stroke | £8,767 | Luengo-Fernandez *et al.,* 2012^2^ |
| Post-stroke (3 months) | £351 | as above |
| Acute TIA | £1,166 | Ward *et al.*, 2007;^3^ NICE guidance, BNF, 2018^4^ |
| Post-TIA (3 months) | £19 | NICE guidance, BNF, 2018^4^ |
| Acute MI | £5,415 | Palmer *et al.,* 2002^5^ |
| Post-MI (3 months) | £240 | Taylor *et al.,* 2009^6^ |
| ACS | £3,249 | Assumed to be 60% of initial costs of myocardial infarction |
| Ongoing ACS (3 months) | £144 | Assumed to be 60% of costs after myocardial infarction |
| Acute stable angina | £409 | An outpatient cardiology assessment plus non-invasive imaging SPECT-CT scan (NHS Reference costs, 2018)^7^ |
| Ongoing stable angina  (3 months) | £6.19 | NICE, 2011,^8^ BNF, 2018^4^ |
| Acute HF (diagnosis) | £3,057 | Griffiths *et al.*, 2014^9^ |
| Ongoing HF | £88 | as above |
| Acute serious fall (hip fracture) | £10,684 | NICE, 2017^10^ |
| Rehab/physical therapy post fall (3 months) | £147 | Polinder *et al.*, 2016^11^ |
| AKI | £6,313 | NHS Reference costs, 2018^7^ |
| Post AKI (3 months) | £1,619 | Hall *et al.,* 2018^12^ |
| Hypotension | £81.00 | 2 GP visits (assumption) |
| Syncope | £101.25 | 2.5 GP visits (assumption) |
| Bradycardia | £121.50 | 3 GP visits (assumption) |
| Electrolyte abnormalities | £111.75 | 2.5 GP visits and 1.5 nurse visits (assumption) |
| Non-serious fall | £40.50 | 1 GP visit (assumption) |

GP=General Practitioner; NICE=National Institute of Health and Care Excellence; MI=Myocardial infarction; TIA=Transient Ischaemic Attack; ACS=Acute Coronary Syndrome; HF=Heart failure; AKI=Acute kidney injury

**Table S2.** Key model assumptions

| Assumption | Justification |
| --- | --- |
| The difference in blood pressure at 12-weeks was assumed to be sustained over patient lifetime. | The was justified in the absence of long-term follow-up data from the trial and tested in sensitivity analyses |
| Treatment safety and efficacy estimates of *deprescribing* were estimated from a meta-analysis of treatment *intensification* trials | There are very few safety and efficacy data from deprescribing studies (very few have been undertaken and have short follow-up). |
| Only one clinical event could occur in a 3-month time cycle. | To limit the complexity of the model and ensure the analysis was feasible. |
| Patient moved to a post-event health state after cardiovascular events and serious adverse events and recurrent events were not modelled. | To limit the complexity of the model and ensure the analysis was feasible. |
| 10-year cardiovascular risk was calculated using the QRisk2 algorithm. | This is used in routine clinical practice in the UK to estimate CVD risk. There are no validated risk scores for patients aged 85 years and over. |
| A multiplier of 1.5 was applied to 10-year cardiovascular risk for patients with existing CVD conditions. | Expert opinion in the absence of any data in this population. |
| Utility values for long-term events and serious adverse effects of treatment were applied multiplicatively to baseline utility scores. | Considered best practice and routinely undertaken in NICE health economic assessments. |
| Disutilities for TIA and minor side effects were assumed to last for one month and were subtracted from utility scores for one 3-month cycle, and disutility for acute kidney disease was applied every cycle over patient lifetime. | Expert opinion in the absence of any data in this population. |

**Table S3.** Sensitivity analysis showing effect varying the relative risk associated with outcome events on the estimated incremental cost effectiveness ratio (ICER)

| Condition | ICER (£ per QALY gained)* | | |
| --- | --- | --- | --- |
|  | **Relative risk=1**  **(no increase risk)** | **Relative risk**  **=lower CI** | **Relative risk**  **=upper CI** |
| Cerebrovascular disease (Stroke/TIA) | £5,753 | £4,216 | £2,846 |
| CHD | £3,010 | £3,657 | £2,580 |
| Heart failure | £9,374 | £4,702 | £2,100 |
| CHD, heart failure, cerebrovascular disease | £178,631 | £17,071 | £1,478 |
| CHD, cerebrovascular disease | £5,882 | £5,956 | £1,822 |
| Serious adverse events | -£784  (usual care dominates) | £7,704 | -£4,656  (usual care dominates) |
| Non-serious adverse events | £2,907 | £3,318 | £2,736 |
| Adverse events and serious adverse events | -£755  (usual care dominates) | £7,971 | -£4,636  (usual care dominates) |

*All ICERs for usual care unless otherwise stated

TIA= transient ischemic attack; CHD=coronary heart disease; QALY=quality adjusted life years; ICER=incremental cost effectiveness ratio

**Table S4.** Sensitivity analyses showing the effect of assuming different effects of blood pressure and absolute cardiovascular risk on the estimated incremental cost effectiveness ratio (ICER)

| Strategy | ICER (£/QALY)* |
| --- | --- |
| Lower 95% CI of BP increase (1mmHg) (Cardiovascular RRs only) | £13,834 |
| Lower 95% CI of BP increase (1mmHg) (All RRs) | £3,189 |
| Absolute risks halved for all cardiovascular events | £4,526 |
| 5-year time horizon | £2,549 |
| Length of time BP difference is sustained |  |
| 10 years | £3,026 |
| 5 years | £3,087 |
| 4 years | £3,207 |
| 3 years | £3,537 |
| 2 years | £3,588 |
| 1 years | £4,159 |

*ICER favours usual care

BP=systolic blood pressure; RR=relative risks;

**Table S5.** Sub-group analyses showing the incremental cost effectiveness ratio (ICER) by baseline frailty and cardiovascular co-morbidities.

| Strategy | ICER (£/QALY)* |
| --- | --- |
| Frailty subgroup  Fit  Frail | £3,397  £2,815 |
| Previous history of cardiovascular disease  None  One condition  Two or more conditions | £4,737  £2,939  £1,801 |

*ICER favours usual care

**Figure S1.** Markov model structure

CHD

Stroke/TIA

Heart failure

Temporary

adverse event

Serious adverse

event

Dead

Well

Straight-line arrows denote movement from one health state to another

Curved-line arrows denote remaining in a health state
